# Effects of pathogenic CNVs on physical traits in participants of the UK Biobank

**DOI:** 10.1186/s12864-018-5292-7

**Published:** 2018-12-04

**Authors:** David Owen, Mathew Bracher-Smith, Kimberley M. Kendall, Elliott Rees, Mark Einon, Valentina Escott-Price, Michael J. Owen, Michael C. O’Donovan, George Kirov

**Affiliations:** MRC Centre for Neuropsychiatric Genetics & Genomics, Institute of Psychological Medicine and Clinical Neurosciences, Cardiff University, School of Medicine, Hadyn Ellis Building, Maindy Road, Cardiff, CF24 4HQ UK

**Keywords:** UK Biobank, CNV, BMI, Waist, Pulse, Weight, Height, Obesity, Anthropometric

## Abstract

**Background:**

Copy number variants (CNVs) have been shown to increase risk for physical anomalies, developmental, psychiatric and medical disorders. Some of them have been associated with changes in weight, height, and other physical traits. As most studies have been performed on children and young people, these effects of CNVs in middle-aged and older people are not well established. The UK Biobank recruited half a million adults who provided a variety of physical measurements. We called all CNVs from the Affymetrix microarrays and selected a set of 54 CNVs implicated as pathogenic (including their reciprocal deletions/duplications) and that were found in five or more persons. Linear regression analysis was used to establish their association with 16 physical traits relevant to human health.

**Results:**

396,725 participants of white British or Irish descent (excluding first-degree relatives) passed our quality control filters. Out of the 864 CNV/trait associations, 214 were significant at a false discovery rate of 0.1, most of them novel. Many of these traits increase risk for adverse health outcomes: e.g. increases in weight, waist-to-hip ratio, pulse rate and body fat composition. Deletions at 16p11.2, 16p12.1, *NRXN1* and duplications at 16p13.11 and 22q11.2 produced the highest numbers of significant associations. Five CNVs produced average changes of over one standard deviation for the 16 traits, compared to controls: deletions at 16p11.2 and 22q11.2, and duplications at 3q29, the Williams-Beuren and Potocki-Lupski regions. CNVs at 1q21.1, 2q13, 16p11.2 and 16p11.2 distal, 16p12.1, 17p12 and 17q12 demonstrated one or more mirror image effects of deletions versus duplications.

**Conclusions:**

Carriers of many CNVs should be monitored for physical traits that increase morbidity and mortality. Genes within these CNVs can give insights into biological processes and therapeutic interventions.

**Electronic supplementary material:**

The online version of this article (10.1186/s12864-018-5292-7) contains supplementary material, which is available to authorized users.

## Introduction

Human height, weight and other anthropometric traits are highly heritable. Genetic factors contribute up to 80% of height [1, 2]. Genome-wide association studies (GWAS) suggest that the total additive effect of single nucleotide variants (SNVs) explain 56% of variance in height and 27% of body mass index (BMI) variability [3]. The heritability of hand grip strength has been estimated at 56% [4]. Resting heart rate, systolic and diastolic blood pressure are also highly heritable, with estimates of 61, 54 and 49% respectively [5].

While SNVs tend to have small effect sizes, large copy number variants (CNVs) have been shown to have profound effects on weight and height, with 16p11.2 deletions and duplications providing striking examples [6]. Other recognised CNVs with large effect sizes on physical traits are deletions at distal 16p11.2, associated with obesity [7], and at 1q21.1, associated with microcephaly and short stature [8]. Failure to thrive has been described in some carriers of 3q29 duplications [9] while short stature has been associated with the 22q11.2 and 22q11.2 distal deletions [10]. Individuals with severe early-onset obesity have an increased rate of large and rare deletions [11]. Obesity can be a feature in rare syndromic monogenic disorders [12], which include several of the CNVs analysed in the current study: 1q21.1 deletion, 15q11-q13 duplication, 16p11.2 and 16p11.2 distal deletions and 22q11.2 duplication.

Most of the published research has been based on children or young people referred for genetic testing for developmental delay, congenital malformations and autism spectrum disorders, i.e. some of the most affected individuals. Such individuals may not be typical of carriers of CNVs, and importantly for long term health outcomes, the impact of these CNVs in middle-aged and older adults (> 40 years), especially in those who have not been diagnosed as CNV carriers in early life, is not well described. In addition, most reports focus on a single, or at most a limited number of CNVs, making it difficult to perform comparative studies of the impact of individual CNVs on physical measures. Another potential problem is that small sample sizes will miss subtle differences in physical traits.

The largest study on CNVs and anthropometric measures was performed on 191,161 unrelated European adults [13]. It assessed systematically the effect of CNVs on BMI, weight, height, and waist/hip ratio in 25 component studies of the Genetic Investigation of Anthropometric Traits (GIANT) Consortium, combined with the first release of UK Biobank data, approximately a third of the total sample. A genome-wide analysis implicated seven CNV regions: 1q21.1 (distal part: 145–145.9 Mb), 3q29 (two sub-regions), 7q11.23 (72.61 72.75 Mb), chr11: 26.97–27.19 Mb; 16p11.2 distal, 16p11.2; chr18: 55.81–56.05 Mb (Mb intervals in hg18). These loci were associated at genome-wide significance with at least one of the four traits in a mirror effect model in which deletions and duplications affect the trait in opposite directions. No common CNVs were significantly associated with the traits, despite the higher statistical power to detect such associations.

Here we report an analysis of the full UK Biobank cohort. We tested 54 CNVs that have been proposed to be pathogenic and were carried by at least five participants. We analysed these CNVs for association with a set of 16 physical traits (Table 1 and Methods), that include the anthropometric traits analysed by the GIANT Consortium (weight, height, BMI, waist/hip ratio), as well as other physical traits: pulse rate, blood pressure, arm strength, peak expiratory volume, heel bone mineral density, and the fat percentage of legs, arms and trunk. These traits are associated with adverse health outcomes and increased mortality [14–19].

**Table 1:** List of physical measures from the UK Biobank data included in the analysis. (Details in Methods)

Measure	Biobank Field ID	Procedure	Unit	Number of people tested
Birthweight	20022	Self-report	kg	225,138
weight	21002	Scales	kg	395,584
Height	50	Standing height	cm	395,854
BMI	21001	Weight/height^2^	kg/m^2^	395,441
Hand grip strength right	47	Grip strength device	kg	395,129
Waist circumference	48	Tape measure	cm	396,037
Hip circumference	49	Tape measure	cm	395,992
Waist to hip ratio	48/49	Waist/hip	ratio	395,954
Leg fat percentage right	23111	Bioimpedance	%	389,777
Arm fat percentage right	23119	Bioimpedance	%	389,722
Trunk fat percentage	23127	Bioimpedance	%	389,569
Pulse rate	102	Automated reading	bpm	373,789
BP systolic	4080	Automated reading	mmHg	370,501
BP diastolic	4079	Automated reading	mmHg	370,511
Peak expiratory flow	3064	Spirometry	litres/min	361,464
Heel bone mineral density	3148	Ultrasound bone densitometry	g/cm^2^	228,405

## Results

We tested 16 physical traits (Table 1) for association with 54 CNVs (Methods). This analysis produced 864 phenotype/CNV associations (Additional file 1: Table S1). Of those 75 survive conservative Bonferroni correction for 864 tests (*p* < 5.8 × 10^− 5^). Using the Benjamini-Hochberg’s method, 214 associations were significant at a 10% false discovery rate (FDR) level (marked in bold in Additional file 1: Table S1). Images of the changes in the physical traits associated with each CNV, and their 95% confidence intervals (95%CI), are shown in Additional file 2: Figure S1 and on our institutional website (http://kirov.psycm.cf.ac.uk).

**Fig. 1:**
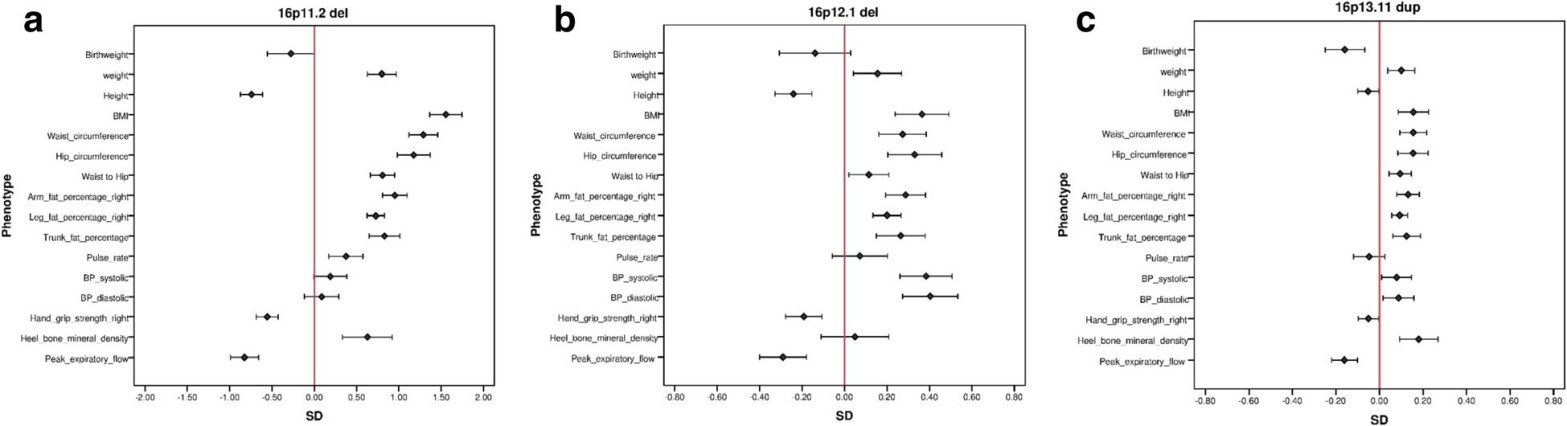
Effect sizes for the physical measurements for the three CNVs with the highest number of significant associations. Effects sizes means are the normalised z-score values (standard deviation changes) from linear regression analysis after correction with co-variates, produced with the *glm* function of R. The figures show the mean changes in z-scores and the 95% confidence intervals of the changes. a) 16p11.2 deletion, b) 16p12.1 deletion, c) 16p13.11 duplication

Table 2 summarises the significant findings. The table is restricted to CNVs that have at least one significant association at FDR = 0.1, with the direction of the effect indicated with + or -. To simplify the presentation, we grouped together the three fat percentage measures to indicate any change on arm, leg or trunk fat % measures and we don’t show the waist and hip circumferences, as the waist/hip ratio reflects this information. Figure 1 (a-c) shows the distribution of changes for the three CNVs with the largest number of significant associations (13 each): 16p11.2 deletion, 16p12.1 deletion and 16p13.11 duplication. The significance also depends on sample size, so these are not necessarily the most pathogenic CNVs. A better measure for overall pathogenicity could be the average absolute effect size, which does not depend on the sample size (Table 2, last column). Five CNVs produced an average effect size change of over one SD for the 16 phenotypes: deletions at 16p11.2 and 22q11.2, and duplications at 3q29, the Williams-Beuren and Potocki-Lupski regions.

**Table 2:** CNVs with significant associations at FDR = 0.1 for physical traits. “+” or “-” indicate the direction of the change. Fat % indicates a significant change on any one of arm, leg or trunk fat % measurements. The last column shows the average absolute difference (normalised coefficients from linear regression) of the 16 tests for each CNV

CNV	Weight	Height	BMI	Hand grip strength	Fat %	Pulse rate	BP	Peak expiratory flow	Heel bone mineral density	Waist/hip ratio	Average difference (B)
TAR del						+					0.37
TAR dup									–		0.24
1q21.1 del	–	–		–				–	–		0.65
1q21.1 dup	+	+			+	+				+	0.24
*NRXN1* del	+		+	–	+			–	–	+	0.25
2q11.2 del	+		+		+				+	+	0.75
2q13 del	+		+	–	+			–			0.52
2q13 dup	–	–	–					–			0.38
2q13dup *NPHP1*				–							0.04
2q21.1 dup				–							0.20
3q29 del		–		–		+	+				0.99
3q29 dup	+		+		+						1.77
WBS dup				–				–	+	+	1.12
7q11.23dup distal							+	–			0.55
13q12.12 dup	–	–			–						0.17
13q12del *CRYL1*		+									0.12
15q11.2 del		–	+		+		+	–	+	+	0.16
15q11.2 dup			+	–	+						0.05
PWS dup				–				–		+	0.71
15q11q13 del BP3 BP4			+					–			0.83
15q11q13 dup BP3 BP4					+						0.29
15q13.3 del	+							–		+	0.50
15q13.3 dup		–		–	+	+	+				0.29
15q13.3 del *CHRNA7*								–			0.67
15q24 dup			+		+						0.76
16p11.2 del	+	–	+	–	+	+		–		+	1.56
16p11.2 dup		+	–	–	–		–		–		0.23
16p11.2 distal del	+		+		+					+	0.71
16p11.2 distal dup	–	–	–	–	–			–	–		0.36
16p12.1 del	+	–	+	–	+		+	–		+	0.38
16p12.1 dup					–						0.11
16p13.11 del				–				–			0.20
16p13.11 dup	+		+		+		+	–	+	+	0.16
17p12del HNPP				–		+	+				0.40
17p12dup CMT1A		–		–	+		–	–	–		0.93
Potocki Lupski		–		–	+						1.00
17q12 del				–		+					0.71
17q12 dup						–					0.25
22q11.2 del		–		–	+	+		–	–	+	1.35
22q11.2 dup	+	–	+	–	+					+	0.21

### Mirror phenotypes

**Fig. 2:**
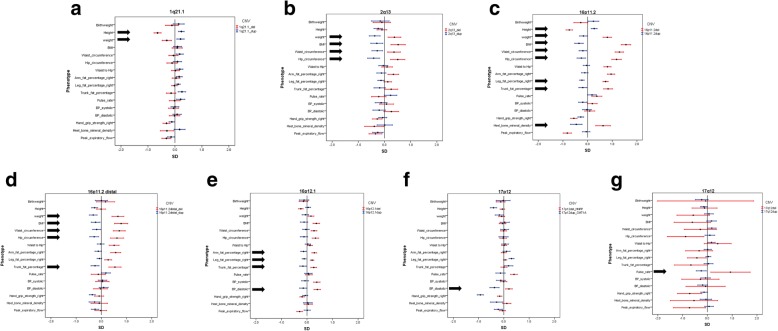
Effect sizes for the physical measurements for the seven CNVs with significant “mirror phenotypes”, marked with arrows: **a**) 1q21.1: height and weight, **b**) 2q13: weight, BMI, waist and hip circumference, **c**) 16p11.2: height, weight, BMI, waist and hip circumference, leg and trunk fat percentage, and heel bone mineral density **d**) 16p11.2 distal: weight, BMI, waist and hip circumference and trunk fat percentage, **e**) 16p12.1: arm, leg and trunk fat percentage, diastolic blood pressure, **f**) 17p12: diastolic blood pressure, **g**) 17q12: pulse rate

A number of reciprocal deletions/duplications at the same locus show significant differences in opposite directions. We decided to use a simple definition for a CNV that produces a mirror phenotype: at least one measure should be changed in opposite directions, both significantly different from controls at FDR = 0.1. Using this definition, we find seven mirror image CNVs: 1q21.1, 2q13, 16p11.2, 16p11.2 distal, 16q12.1, 17p12 and 17q12 (Fig. 2 a-g). Inspection of the directions of the effects of all CNVs (Additional file 2: Figure S1) suggests that more CNVs might produce genuine mirror phenotypes, if tested in larger samples. Our results confirm three of those reported by Macé et al. [13],: 16p11.2, 16p11.2 distal and 1q21.1, while 3q29 also suggests multiple mirror phenotypes in our data (Additional file 2: Figure S1), but none of them reached significance, most likely due to the small number of observations (9 deletions and 5 duplications). The other four findings by Macé et al. could not be tested in our data: in the 7q11.23 region we found only one deletion and the 11p14.2 and 18q21.32 regions were not on our list. Here we report associations on 12 more physical traits and identified four more significant loci. Further comparisons with physical, social and mental health measures were reported by Macé et al. [13] for two CNVs: 16p11.2 and 16p11.2 distal, affirming the high pathogenicity of these two loci. Our significant findings for 2q13, 16p12, 17p12 and 17q12 were not reported in the previous study, presumably because some specific measures were not tested in that study (e.g. blood pressure pulse rate, fat %), or due to statistical power issues. We note that although the two studies complement each other, they are not fully independent, as the first third of the UK Biobank sample was also used by Macé et al. [13]. They also differed in the methods used: we tested a pre-defined list of 54 loci, while Macé et al. deployed a genome-wide scan.

## Discussion

### Novel associations

About one quarter of all possible CNV/phenotype associations were significant at FDR = 0.1, suggesting multiple effects on physical traits by many pathogenic CNVs. Some of these associations are already known from previous large case series, e.g. 16p11.2 deletion/duplication and 16p11.2 distal deletion (Introduction), from studies on individuals with syndromic short stature or obesity, and from a large study on 26 cohorts [13], supporting the validity of this dataset and our methods. We show that the majority of these CNVs (41 of the 54) impact on at least one physical trait. Many significant associations have not been reported before in systematically assessed cohorts, and certainly not as part of the same analysis, where identical methods are used, to allow comparisons between CNVs. As examples, we find that deletions in *NRXN1,* a CNV so far known to increase risk for schizophrenia and autism spectrum disorders [20–22], are associated with a plethora of adverse changes, producing 11 significant results, such as increased weight, BMI, waist/hip ratio, fat percentage in arms, legs and trunk, and a faster pulse rate. *NRXN1* carriers also show reductions in muscular strength, peak expiratory flow and heel bone mineral density. Carrier status of 15q11.2 deletions and duplications have not been consistently associated with medical or anthropometric changes, although deletions increase risk for neurodevelopmental disorders [21, 23]. We now show that deletion carriers have significant changes in over half of the traits, most notably reductions in height and birth weight, and increases in bone mineral density and waist/hip ratio. 15q11.2 duplication carriers have increased BMI, fat percentage and waist circumference and reduced muscular strength. The magnitudes of the changes associated with 15q11.2 are small (e.g. deletion carriers are only 1.5 cm shorter in height on average), but the very large sample size of the UK Biobank (~ 1600 deletion and ~ 1900 duplication carriers) allows small changes to be detected with high statistical confidence. Another notable finding is the association of 22q11.2 duplications with 10 measures. Although high rates of psychiatric disorders have been recorded for this CNV [24], due to the extreme phenotypic variability of this condition, its true pathogenicity has been regarded as unclear [25]. We report on a much larger sample (266 carriers) and observe that central obesity (increased BMI and waist-to-hip ratio) is the leading feature in duplication carriers, making this CNV potentially highly pathogenic in relation to health-related outcomes [18, 26].

### CNVs cause adverse effects of likely medical relevance

We note that most (although not all) of the observed effects are likely to be adverse for health. Thus, all 35 significant associations with hand grip strength and peak expiratory flow are in the direction of reduced performance. Reduced hand grip strength and peak expiratory flow are associated with increased mortality [14, 16]. Most associations with pulse rate and blood pressure are in the direction of higher values, indicating a worse cardiovascular performance. Many CNVs cause increased weight/ fat percentage/ central obesity (increased waist/hip ratio), all well-known factors that increase morbidity and mortality [15, 18, 26]. We have indeed shown that in this population, carriers of this set of CNVs have increased mortality and morbidity for many common medical disorders [27]. There are notable exceptions: lower weight (and/or other obesity-related measures) are found in carriers of deletions at 1q21.1 and duplications at 2q13, 13q12.12, 16p11.2 and distal 16p11.2. The potential protective effects of lower weight or fat percentage appear to be offset by other adverse consequences of these CNVs. Thirteen CNVs lead to shorter height, while only three are associated with increased height: 1q21.1 duplications, and deletions at 13q12 (*CRYL1*) and 16p11.2. BMI on its own is not sufficient to assess weight, height and obesity changes. For example, carriers of 1q21.1 deletions are on average 6.0 cm shorter and 4.8 kg lighter, while carriers of the reciprocal 1q21.1 duplications are 2.2 cm taller and 3.3 kg heavier. Despite these substantial changes, both types of carriers have an average BMI. A widely accepted measure of obesity, associated with adverse effect on health outcomes, is the waist/hip ratio, which has been shown to better predict the risk for myocardial infarction [26]. We find this significantly increased in carriers of 13 CNVs, while no CNV caused a significantly reduced ratio, i.e. we do not see any protective effects for medical outcomes, even for CNVs that lead to reduced weight. Our findings that duplications at 15q11.2, 15q13.3, 16p13.3, 22q11.2 and deletions at *NRXN1* are among the CNVs with the highest rates of adverse changes on physical traits, were unexpected and raise important issues regarding the management of such carriers.

### Are the effects primary or secondary?

**Fig. 3:**
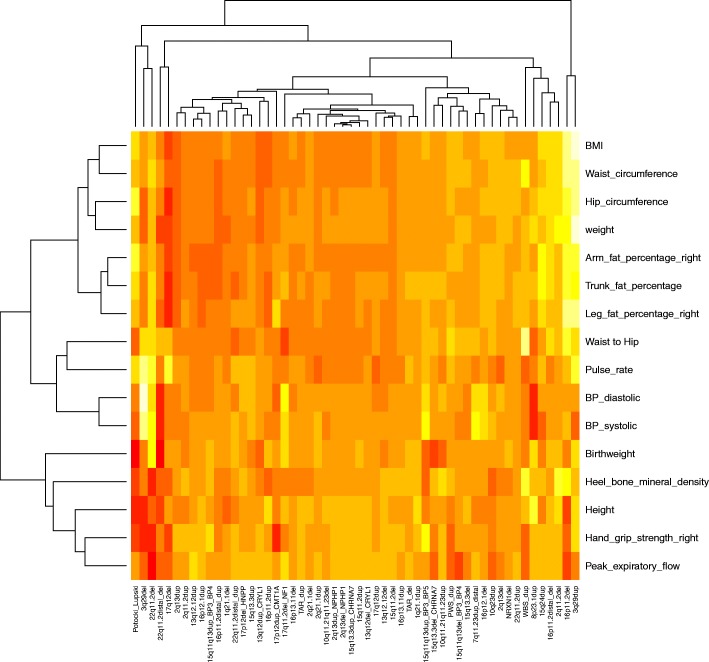
Clustering of the physical measures for all 54 CNVs, with the effect sizes (normalised z-scores) of the 16 associations. Paler colours (yellow to white) indicate a positive direction, while darker red colours show a negative direction of effect

Some of the changes in physical traits could be due to life-style differences among CNV carriers, or to medication given for diseases caused by the CNVs, rather than to a direct biological effect from gene dosage changes. Current knowledge of CNV effects are largely restricted to childhood, while the UK Biobank population is composed of middle-aged and older adults (ranging between 40 and 69 years old at recruitment), a difference that may result in lifestyle-related effects in this cohort. Reduced muscular strength, lower peak expiratory flow, faster pulse rate, increased weight and fat percentage can all be consequences of reduced exercise and life-style changes. Having a pathogenic CNV might make the person less likely to exercise, due to medical, cognitive or social problems. However, it appears that lifestyle is unlikely to account for all the changes we report. Thus, it cannot explain why at least seven CNV loci have opposite (mirror) phenotypes in deletion and duplication carriers (Fig. 2) and why four CNVs lead to reduced weight (1q21.1 deletion, 2q13 duplication, 13q12.12 duplication and 16p11.2 distal duplication). The 1q21.1 duplication carriers present with increased height and weight, which together with the macrocephaly reported in children with this duplication [8] suggests that this is an overgrowth syndrome [28], although with a variable expressivity [8]. Such observations indicate that many of the differences are a direct consequence of gene dosage changes, rather than just secondary to life-style or social factors. In fact, each CNV has its own unique signature of physical traits, which becomes apparent on a heatmap image (Fig. 3). Having a diagnosis of a neurodevelopmental disorder (with the resulting medication intake and life-style changes) can not account for the observed changes either. Only 1179 persons in the tested sample (0.3%) had a diagnosis of “mental retardation”, autism or schizophrenia. This low rate is due to the recognised “healthy volunteer” selection bias that operated during the UK Biobank recruitment, resulting in this population being less socioeconomically deprived, and having lower morbidity and mortality than the general population [29]. We re-analysed the data after excluding these 1179 people and present the comparison of the results in Additional file 3: Table S3. Out of the 214 results originally significant at FDR = 0.1, only 8 lost their significance, while a similar number of new ones reached significance. The effect sizes remained essentially identical, apart from some fluctuations on three of the rarest CNVs where a high proportion of carriers were diagnosed with neurodevelopmental disorders: two out of nine carriers of 3q29 deletions had schizophrenia, and two cases of “mental retardation” were found in both Potocki-Lupski Syndrome duplications (out of five carriers) and 22q11.2 deletions (out of 10 carriers). This is best captured on a scatterplot included in Additional file 3: Table S3, where the outliers belong largely to these three CNVs.

## Conclusions

Our work provides an unbiased comparison of physical measures between adult CNV carriers, as all Biobank participants were assessed with the same methods and blindly to their CNV status. Our findings of adverse changes in basic physical characteristics indicate that carriers of these CNV could benefit from general monitoring for cardiovascular risk factors. It is tempting to envisage the targeting of genes or biochemical pathways in order to improve weight or fat distribution in humans. From this perspective, the CNVs with the most pronounced “mirror” phenotypes (Fig. 2) are likely to contain the most promising candidate genes, as gene dosage changes lead to reciprocal changes in the measurements.

## Materials and methods

### CNV calling

We downloaded from the UK Biobank the raw intensity (CEL) files from Affymetrix BiLEVE (N~ 50,000) and Axiom (N~ 450,000) arrays and processed them with Affymetrix Power Tools (www.affymetrix.com/estore/partners_programs/programs/developer/tools/powertools.affx) and PennCNV [30]. We followed the same CNV calling pipeline that we described previously [31].

### Criteria for choosing CNVs for analysis

We called a list of 92 CNVs proposed to be pathogenic in two widely accepted sources [23, 32]. These lists included CNV regions that lead to genomic disorders or clinically significant phenotypes, reported in databases or multiple publications (Additional file 4: Table S2). Most of them have been shown to statistically increase risk for developmental delay [23]. The table lists the 92 CNVs and the reasons for exclusion or retention in the analysis. The reciprocal deletions/duplications of known genomic disorders were also included in the source publications and in the current study, even if the evidence for their pathogenicity in unclear, in order to study potential mirror phenotypes and so-far unconfirmed pathogenicity. We excluded CNVs with fewer than five observations, and three small CNV loci that produced calls predominantly on arrays with poor QC, i.e. likely to generate false-positive calls. This left 54 CNVs suitable for analysis.

### Physical traits

We obtained data from the UK Biobank on tests which were performed at the assessment centres and described as “physical measurements” (biobank.ctsu.ox.ac.uk/crystal/docs/Bodycomposition.pdf). These include anthropometric measures (height, weight, BMI, hip and waist circumference), body fat content, hand grip strength, spirometry, ultrasound heel bone densitometry and self-reported birthweight (Table 1). Body fat content is estimated from the bioimpedance measures performed with a Tanita BC418MA body composition analyser. We also included pulse rate and blood pressure, as recorded at the assessment centres. In order to maximise statistical power and simplify the presentation of the results, we only used tests collected on > 50% of individuals and excluded variables correlated at > 0.9, such as measures collected on left and right arm or leg. We therefore only analysed measures performed on the right arm or right leg and averaged the two pulse rate measures that were performed at the same initial visit. Following research that highlights the importance of the waist to hip ratio [26], we added this measure too, resulting in a set of 16 variables.

### Statistical analysis

We filtered out poorly performing arrays using the following cut-off criteria: genotyping call rate < 0.96, > 30 CNVs per person, a waviness factor of < − 0.03 & > 0.03 & LRR standard deviation of > 0.35. We excluded people who self-report to be other than white British or Irish, and first-degree relatives (using the kinship coefficients data). This left 396,725 people for analysis. The tested variables followed normal distributions, therefore we did not further transform the data prior to analysis, other than to normalise all measures into z-scores, for a uniform presentation. We used linear regression analysis (glm) in R (version 3.3.2) to test the effect of the CNV carrier status on each measure (in z-score differences). We used the same set of co-variates for all associations: sex, age, array type (Axiom/BiLEVE), Townsend deprivation index (as a measure of the socioeconomic status) and the first 15 principal components from the genetic analysis, as provided by the UK Biobank. We also provide the changes in non-normalised (original) units, to give a more real-world view of the effect of CNV carrier status (e.g. kg, beats per minute, mmHg). We did not control for education or occupation, as we have shown that these are likely to be consequences of CNV carrier status [31]. A Bonferroni correction for 864 test gives a level of significance of *p* < 5.8 × 10^− 5^, which is conservative, given many of the measures are correlated (e.g. BMI and waist/hip ratio). As many true associations were expected, it is more appropriate to use the Benjamini-Hochberg false-discovery rate (FDR) method [33]. We accepted FDR = 0.1 as our significance threshold, reasoning that 10% of false positives is a reasonable trade-off for this type of analysis (Additional file 1: Table S1).

## Additional files

Additional file 1: **Table S1.** All results from linear regression analysis. Separate tables are provided for each CNV. (DOCX 1040 kb) (XLSX 335 kb)

Additional file 2: **Figure S1.** Images of the changes in the physical traits (normalised z-score values) associated with each CNV and their 95% confidence intervals (95%CI). (XLSX 335 kb) (DOCX 1040 kb)

Additional file 3: **Table S3.** Comparison of all associations before and after the exclusion of 1179 people with a neurodevelopmental diagnosis: schizophrenia, autism and “mental retardation”. “new” refers to results obtained after the exclusion of these people. The scatter plot compares the normalised z-score values form linear regression analysis before and after the exclusions. The few outliers are generated from three CNVs: 3q29 deletions, Potocki-Lupski Syndrome duplications and 22q11.2 deletions where a disproportionate number of carriers had neurodevelopmental disorders (details in Discussion). (XLSX 14 kb) (XLSX 197 kb)

Additional file 4: **Table S2.** List of the 92 CNVs considered for analysis. “Significant (Coe, 2014)” indicates the CNVs that have been shown to be significantly associated with developmental delay, autism spectrum disorders or multiple congenital anomalies in that study [23]. “Genomic disorder (Dittwald et al, 2013)” indicates the CNVs included in the study by Dittwald et al. [32] as implicated in genomic disorders or clinically significant phenotypes. “Unreliable” indicates those CNVs (mostly telomeric) that produced calls predominantly on arrays that failed QC. This indicates that they could generate false-positive calls even on arrays that pass QC and therefore were excluded from analysis. “Rare” CNVs are those with < 5 observations, also excluded from analysis. (XLSX 197 kb) (XLSX 14 kb)

## Abbreviations

BMI: Body Mass Index
CNV: Copy Number Variant
FDR: False Discovery Rate
