# Supplementary material for: Effects of pathogenic CNVs on physical traits in participants of the UK Biobank
Source: BMC Genomics. 2018 Dec 4;19:867. doi: 10.1186/s12864-018-5292-7 (PMC6278042; doi:10.1186/s12864-018-5292-7)

54 images demonstrating the direction of effect for each anthropometric measure as a function of change in standard deviation. Error bars represent 95% confidence intervals.


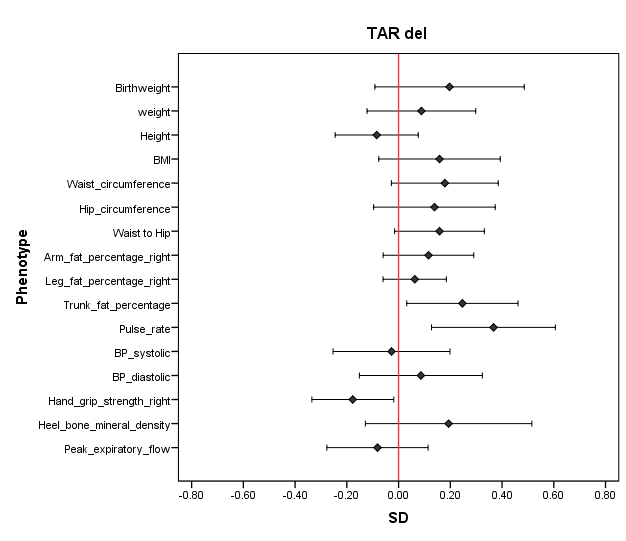

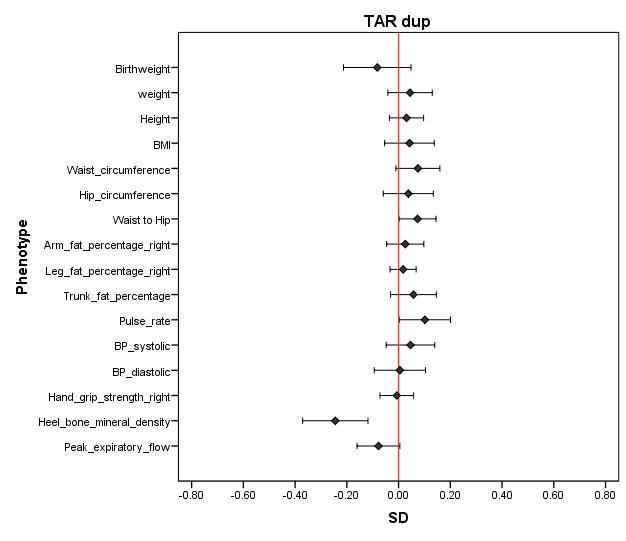


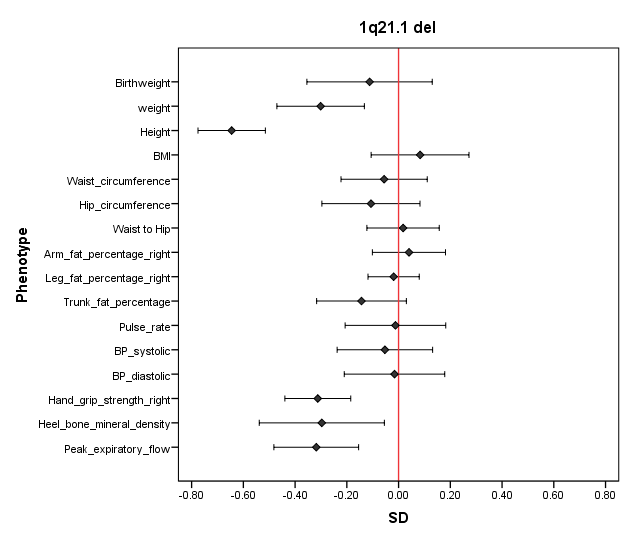


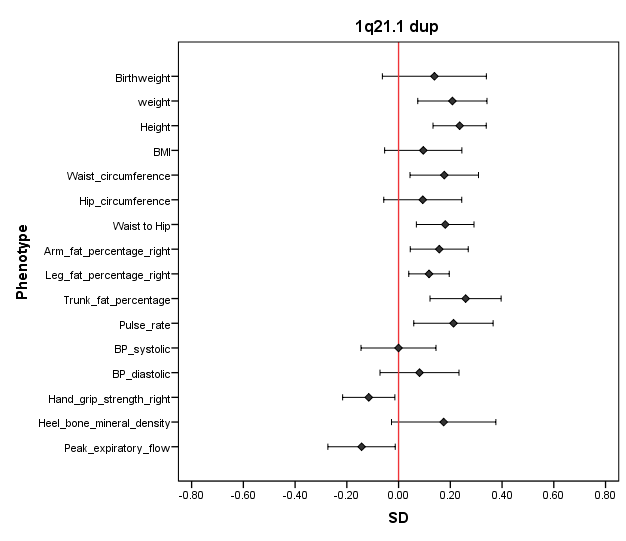


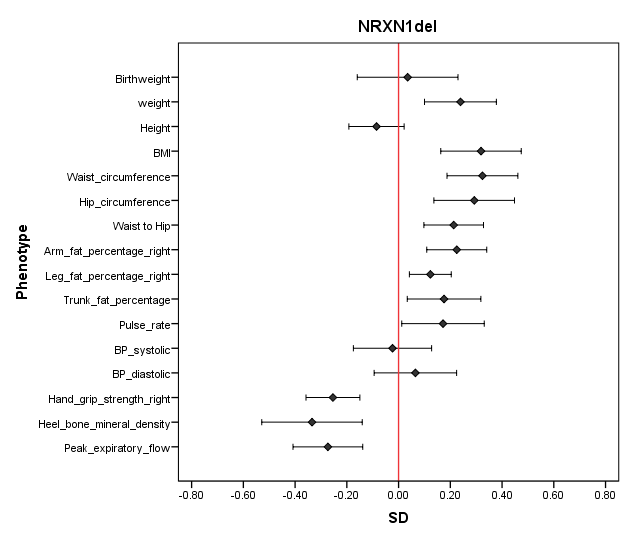


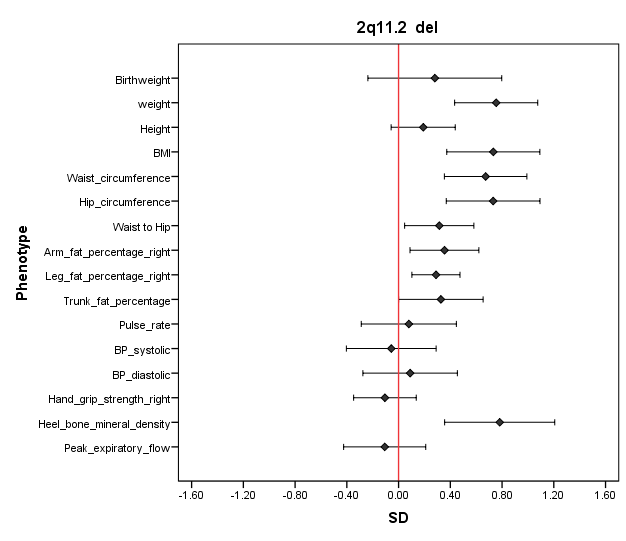

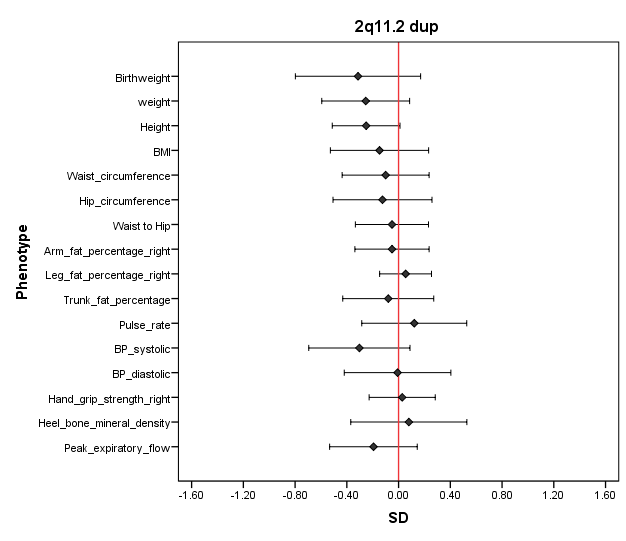


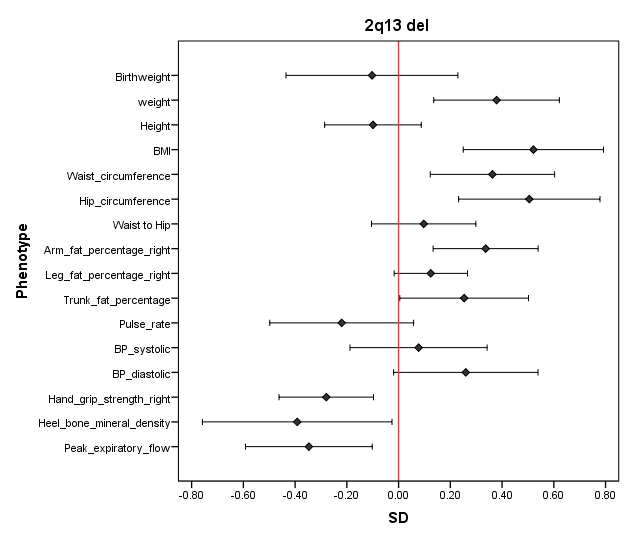

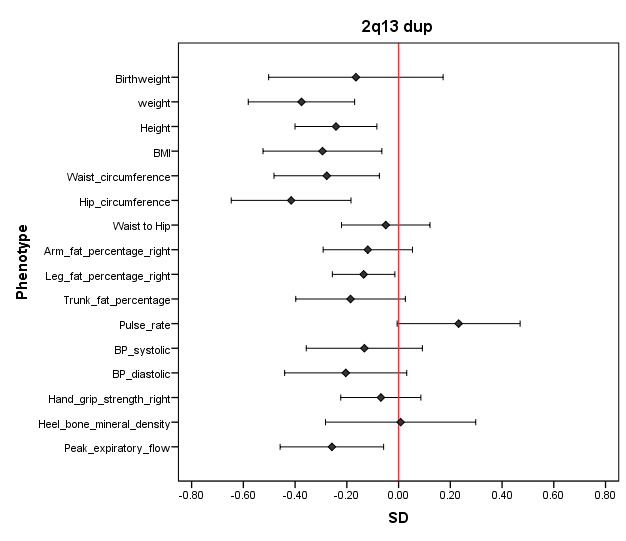


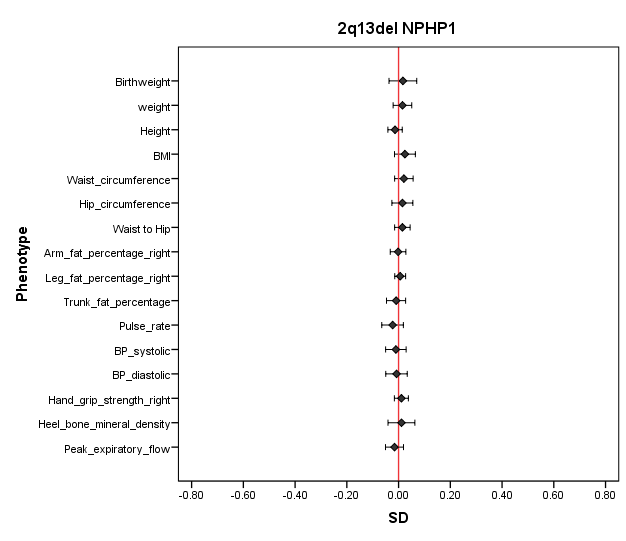

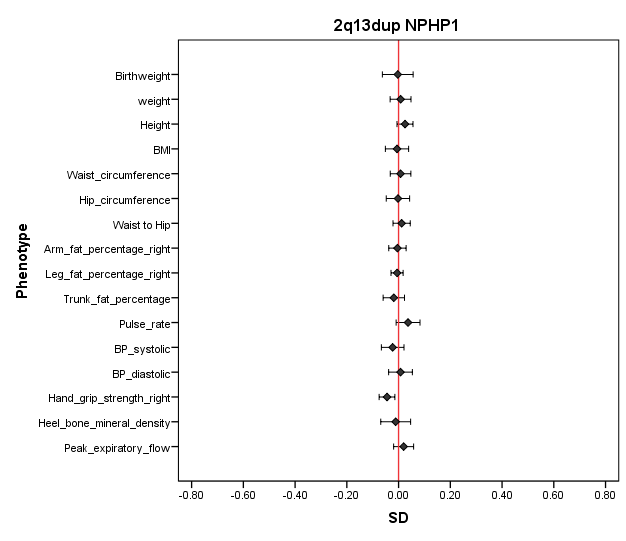


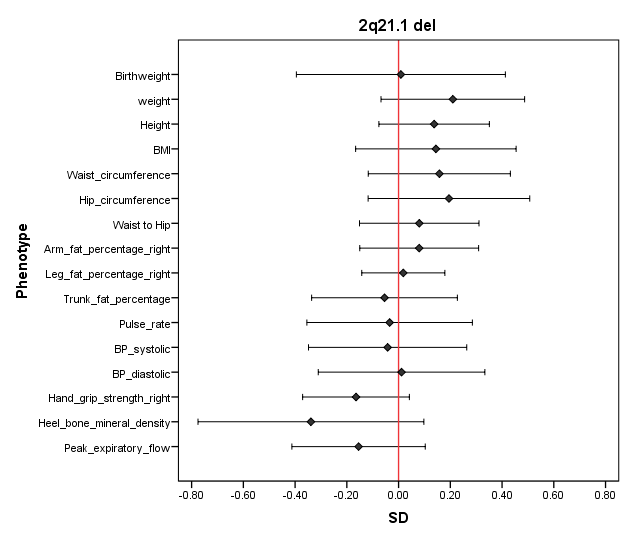

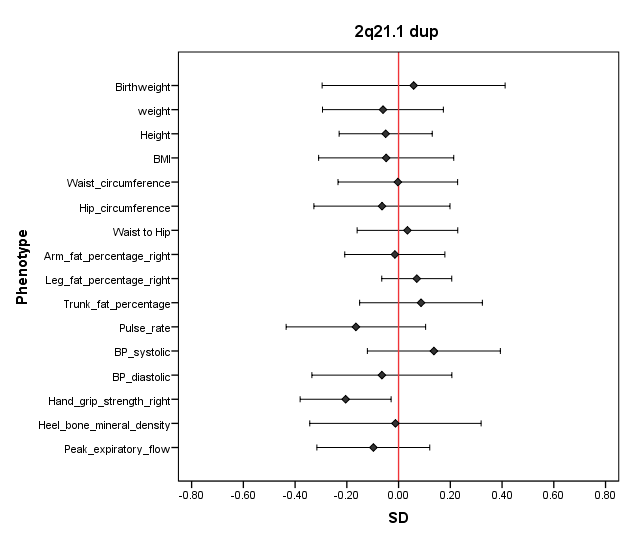


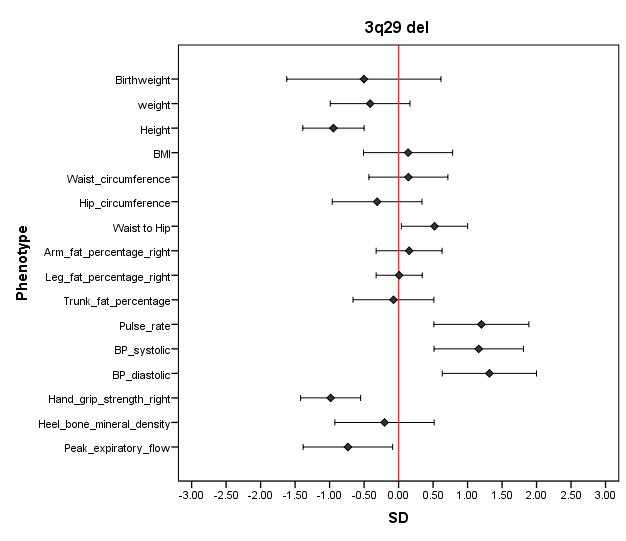

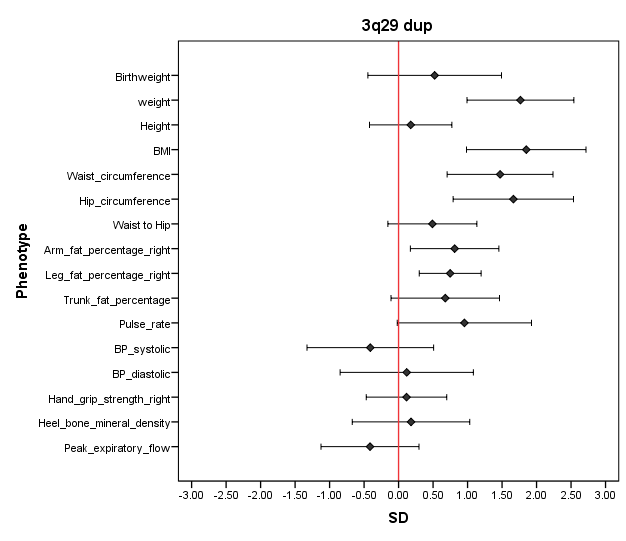


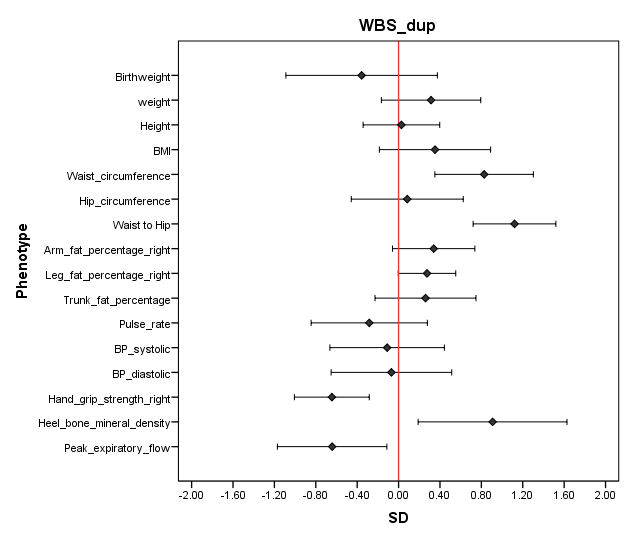


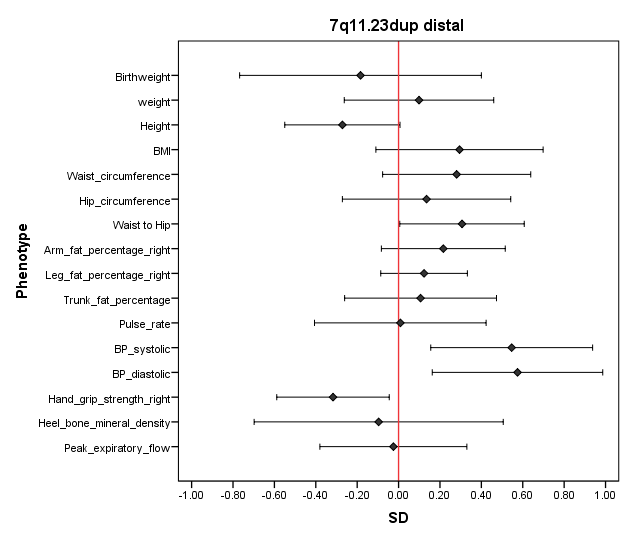


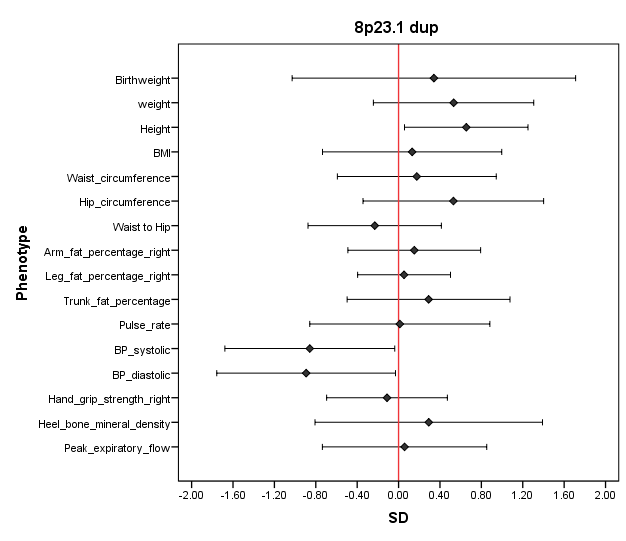


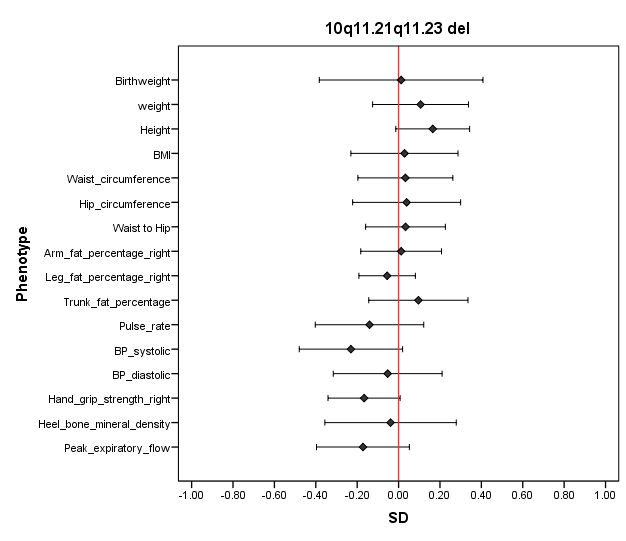

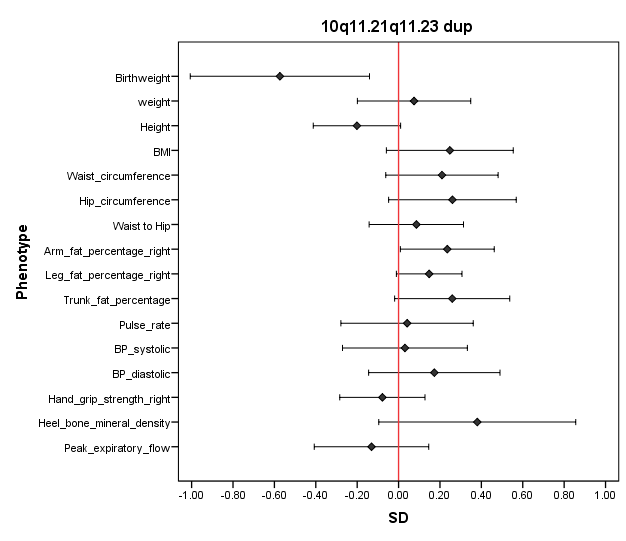


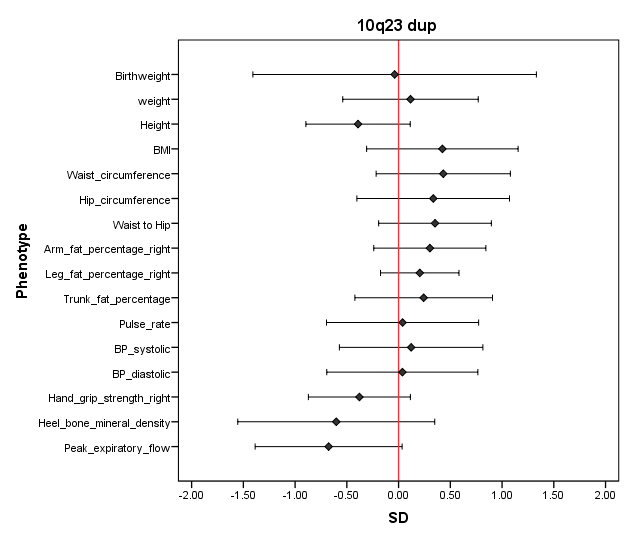


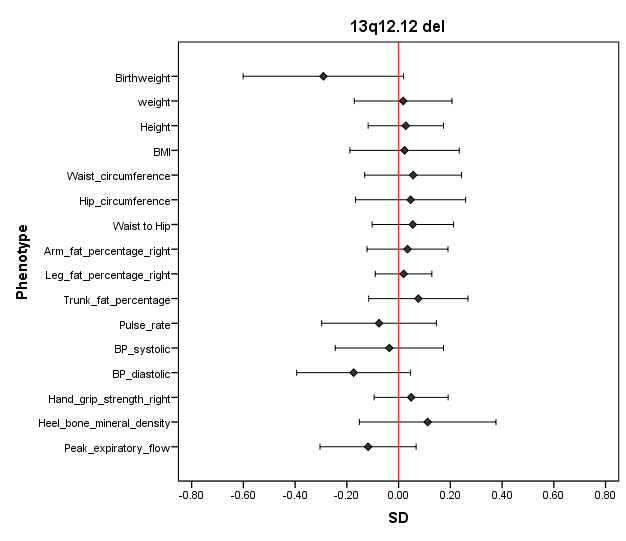

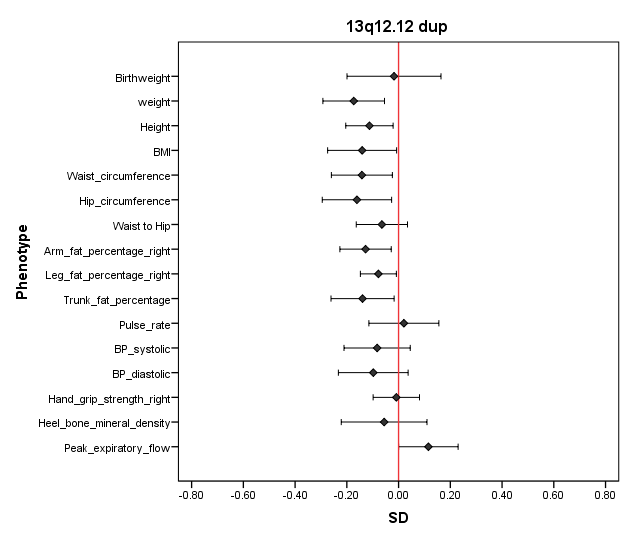


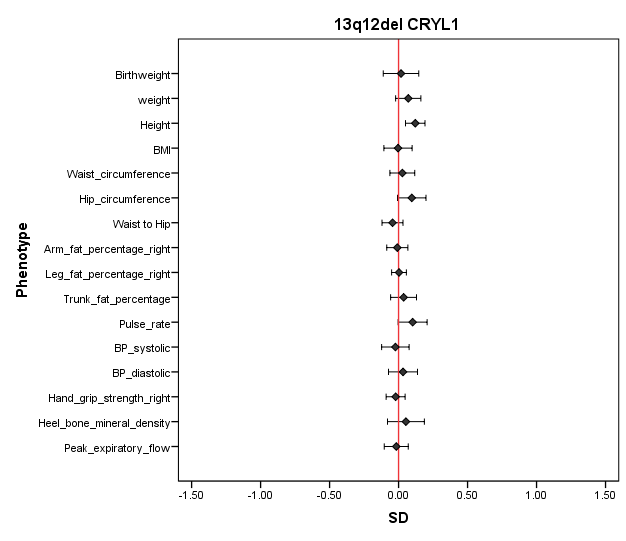

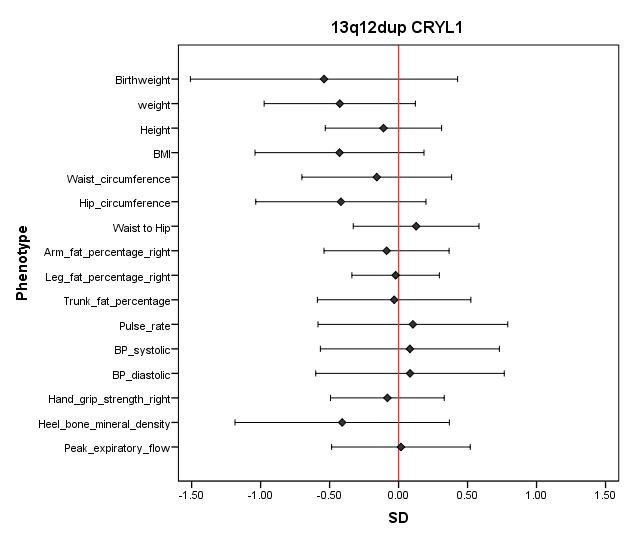


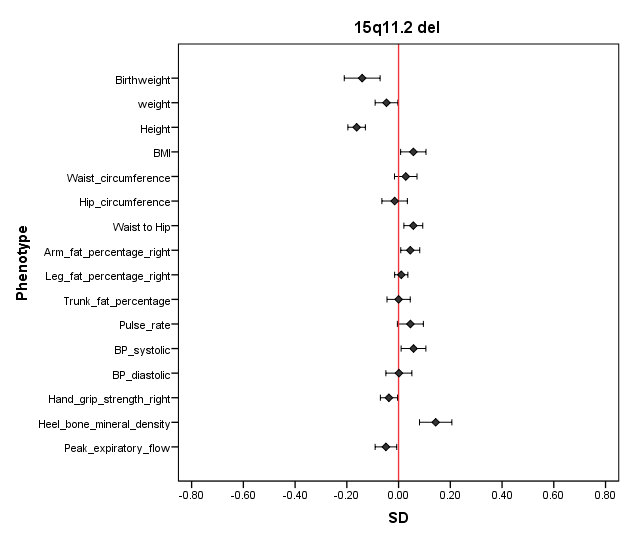

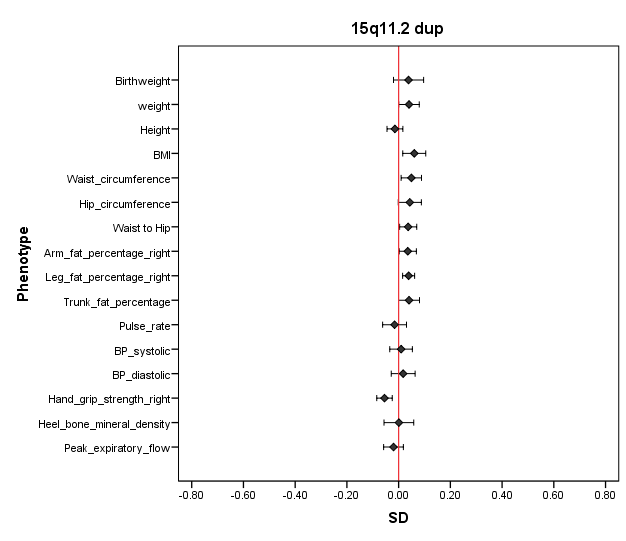


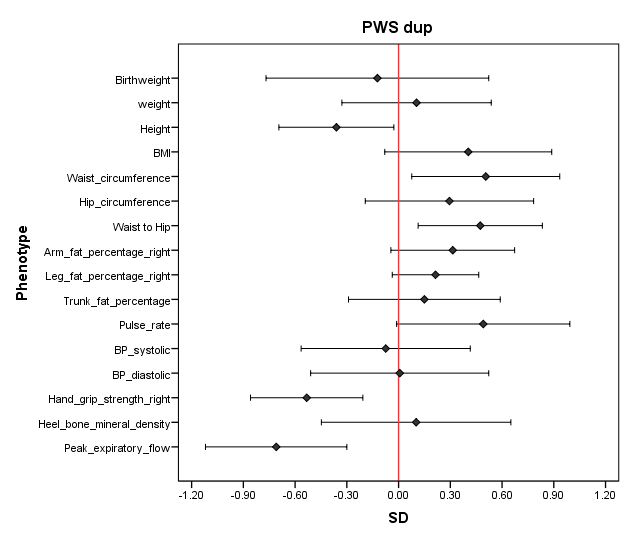


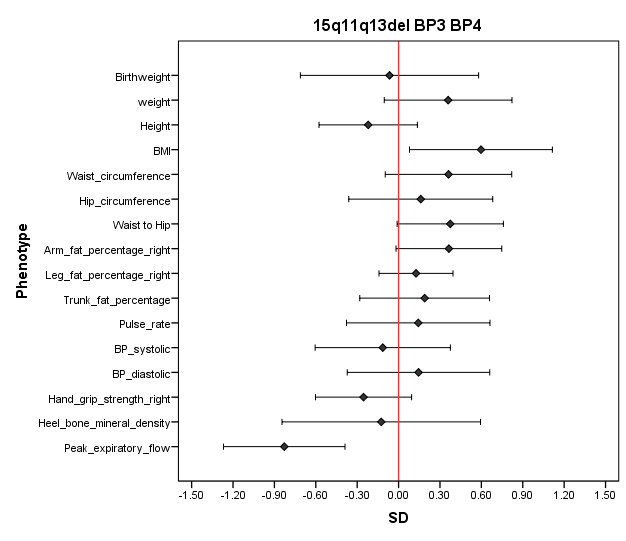

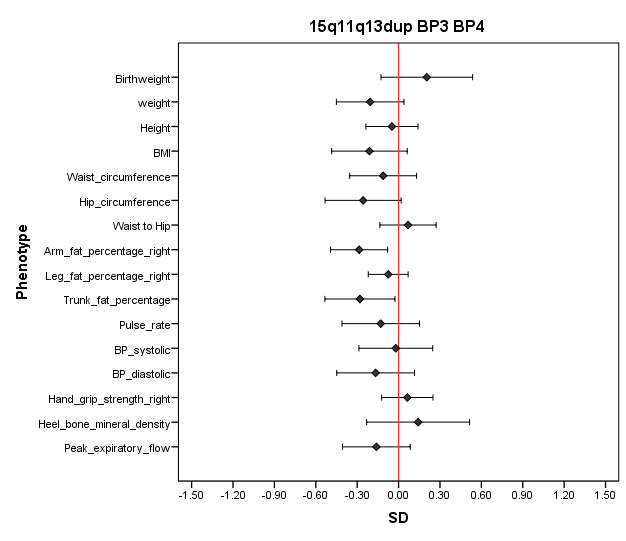


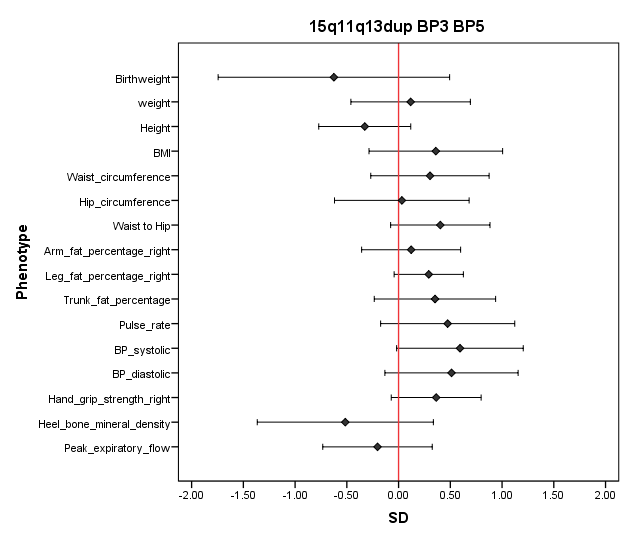


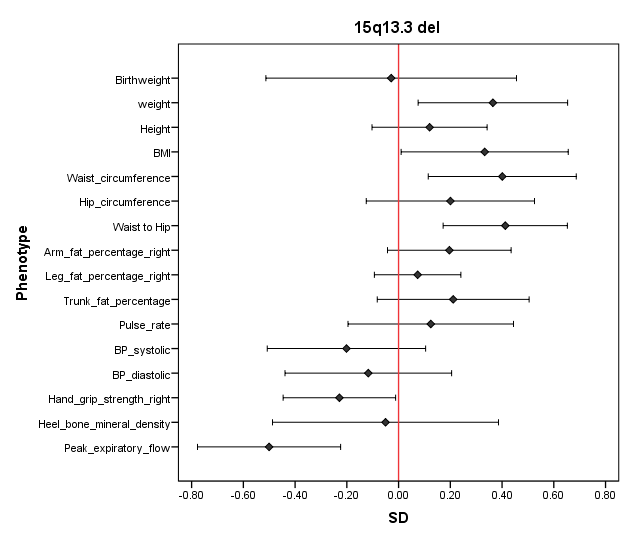

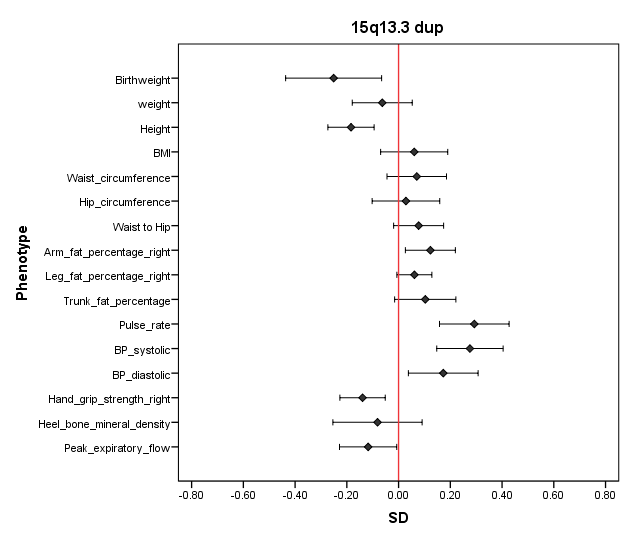


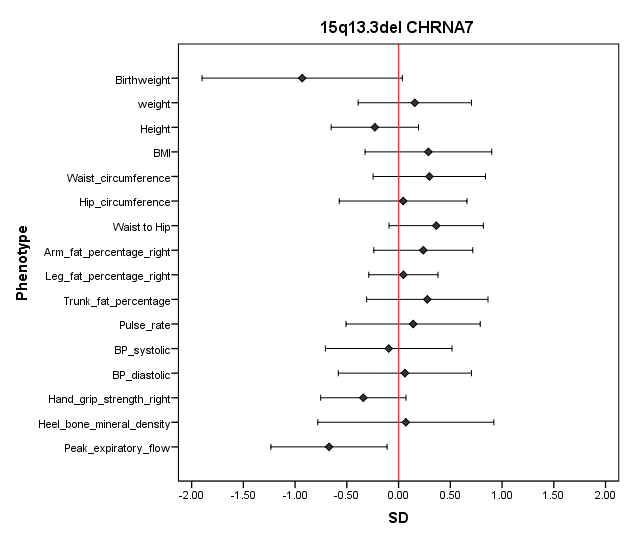

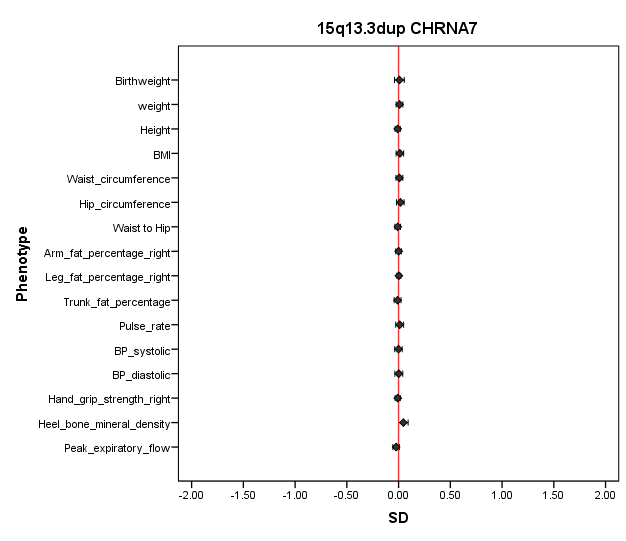


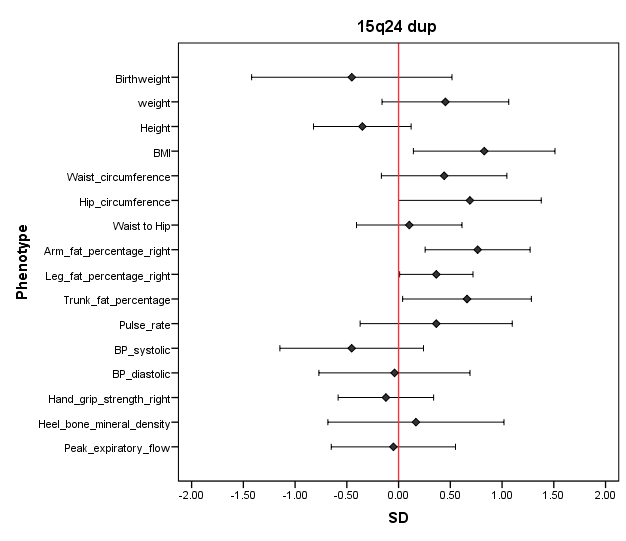


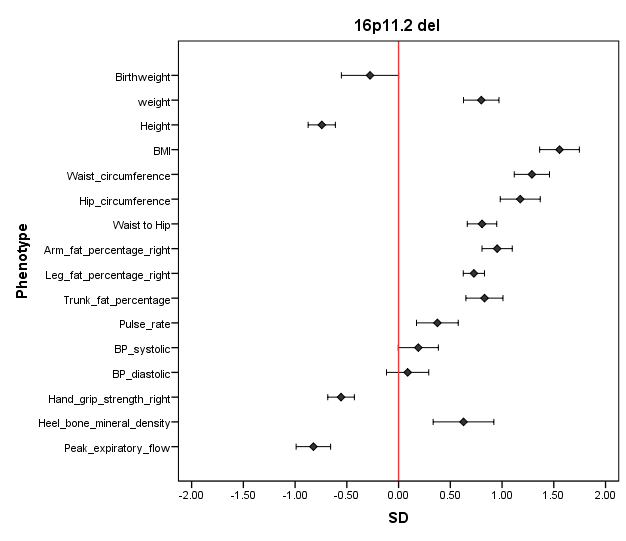

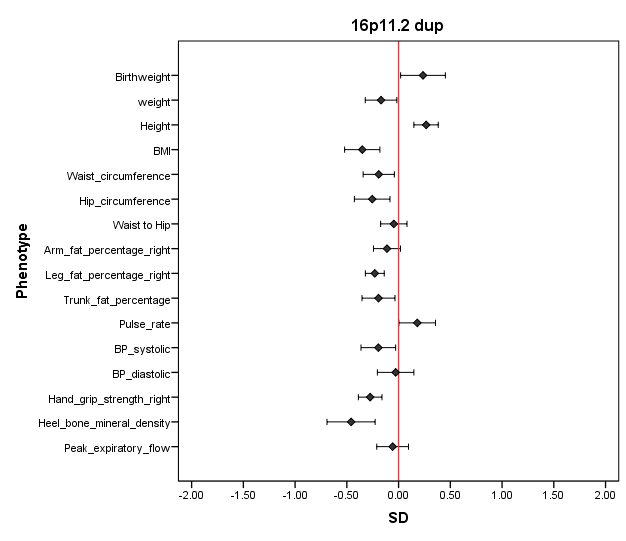


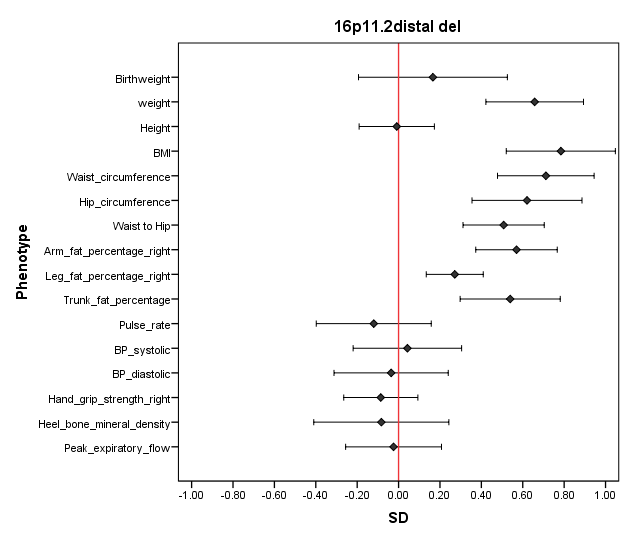

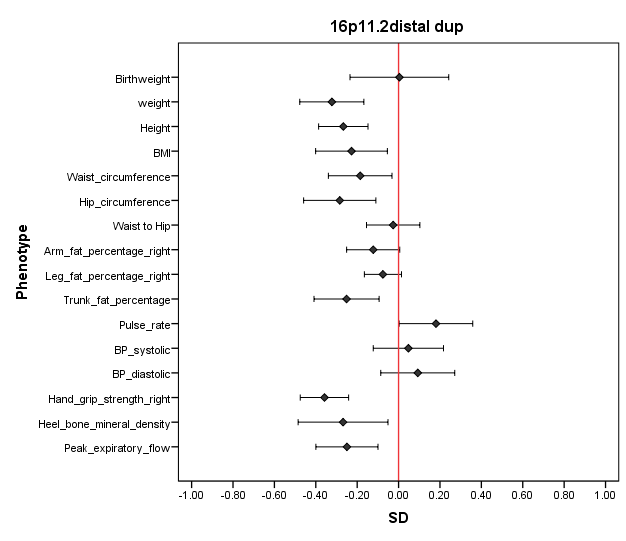


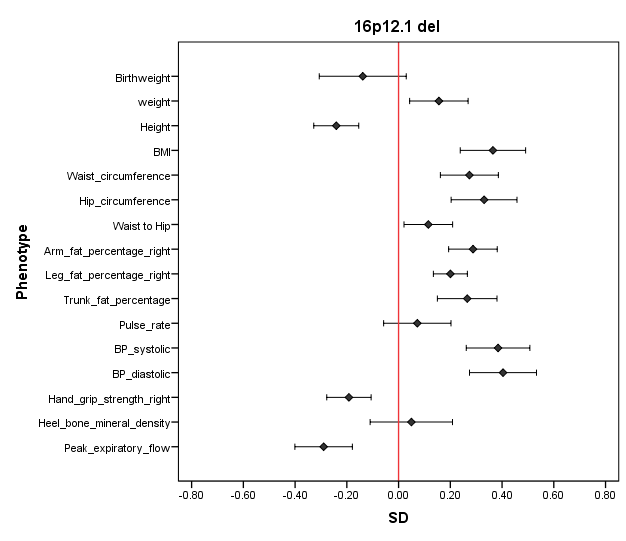

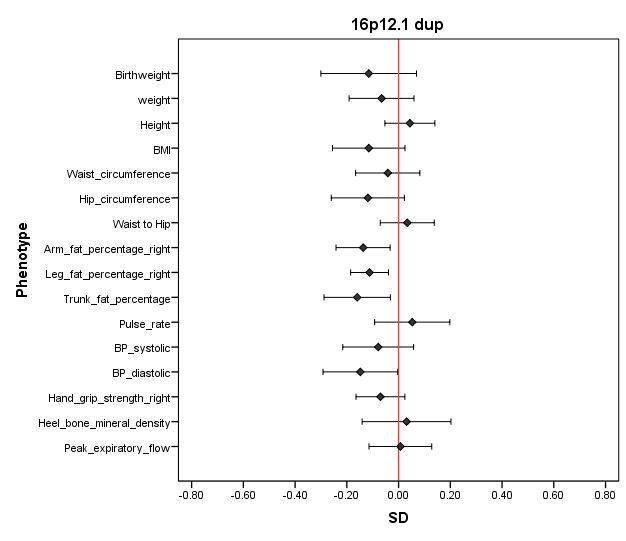


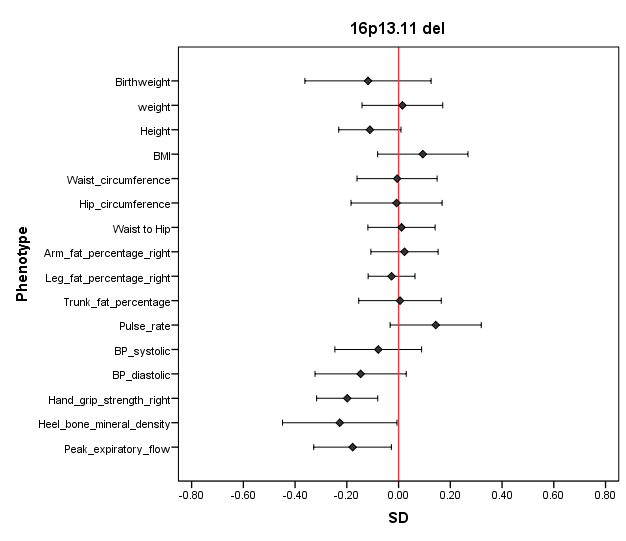

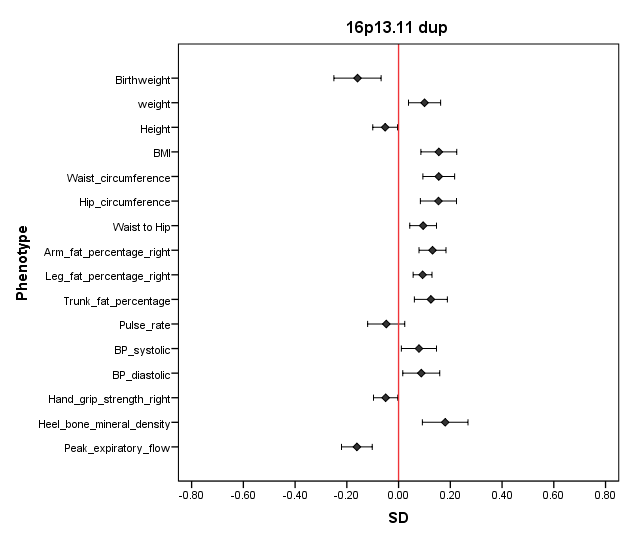


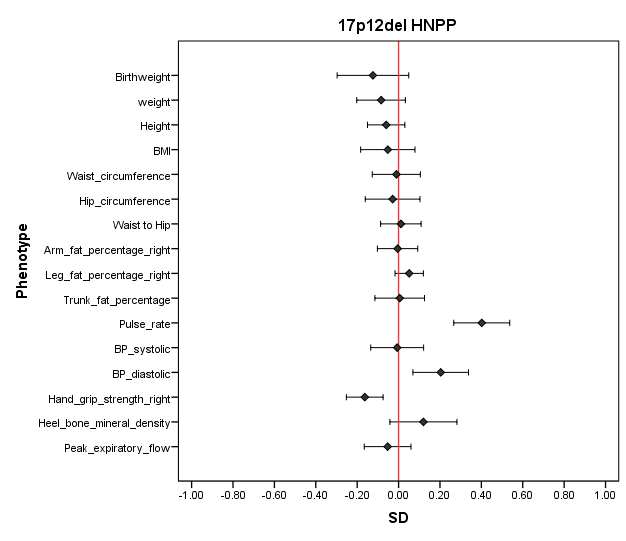

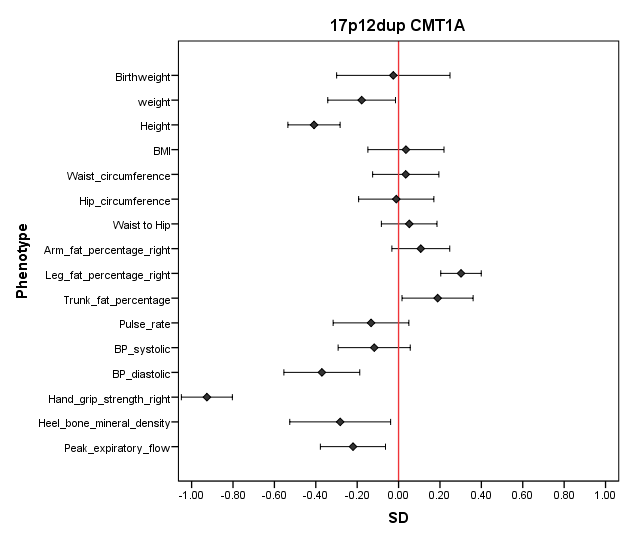


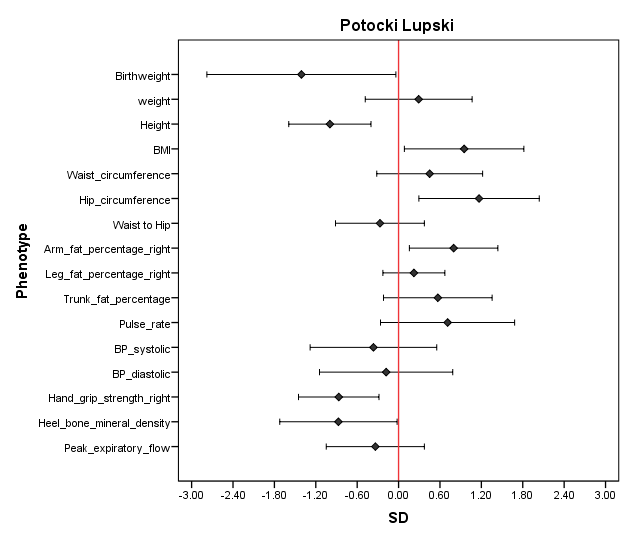


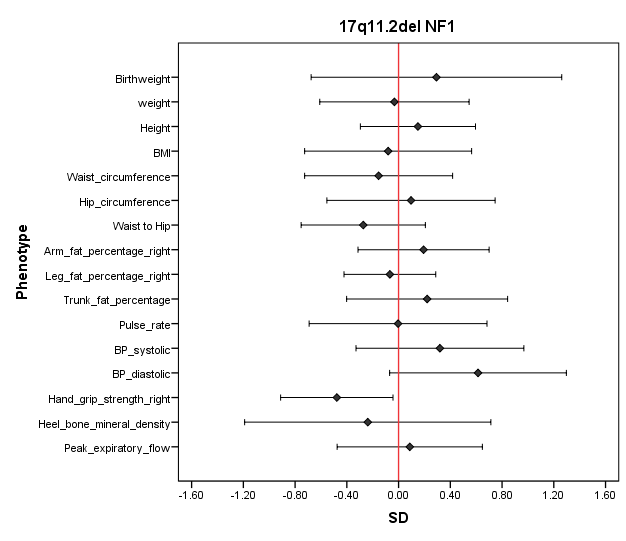


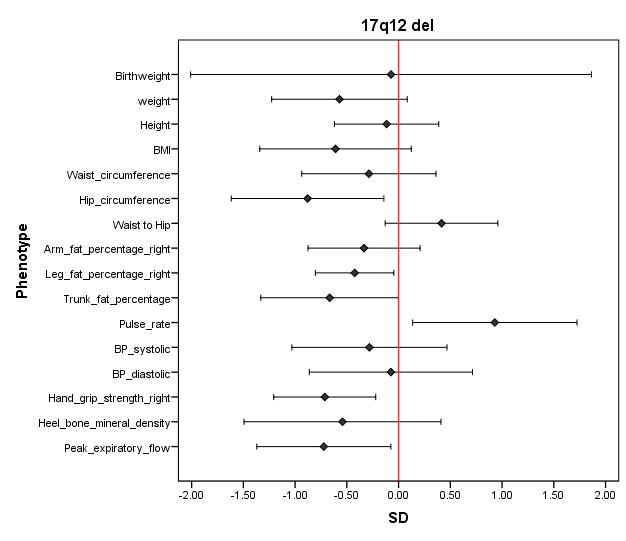

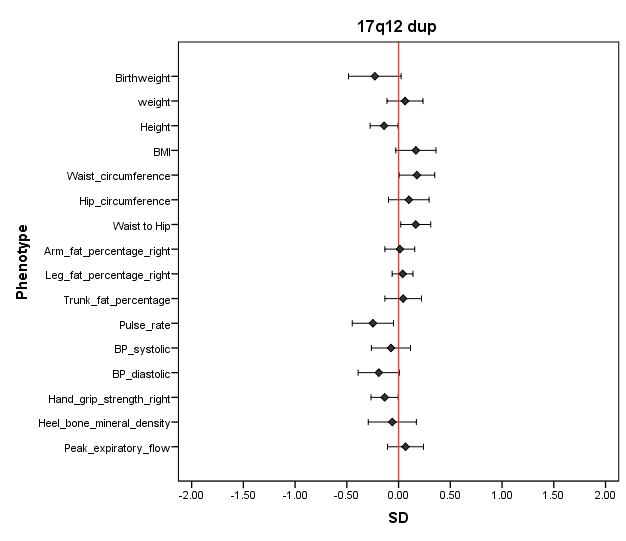


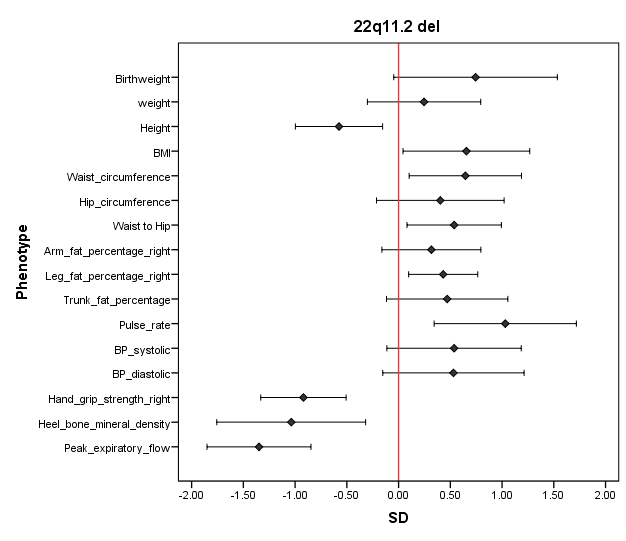

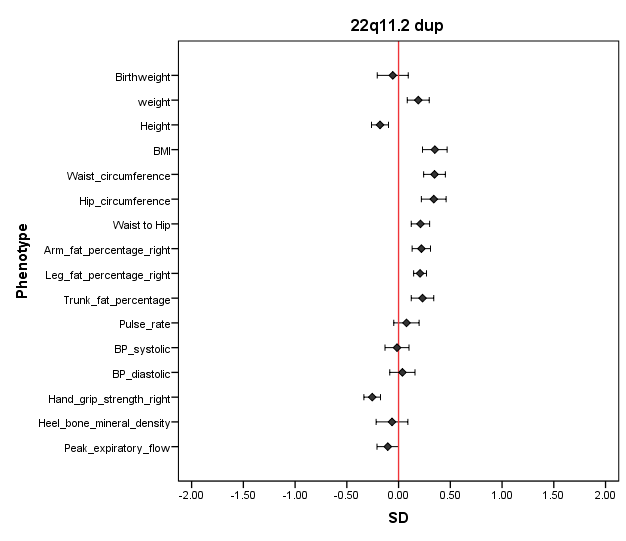


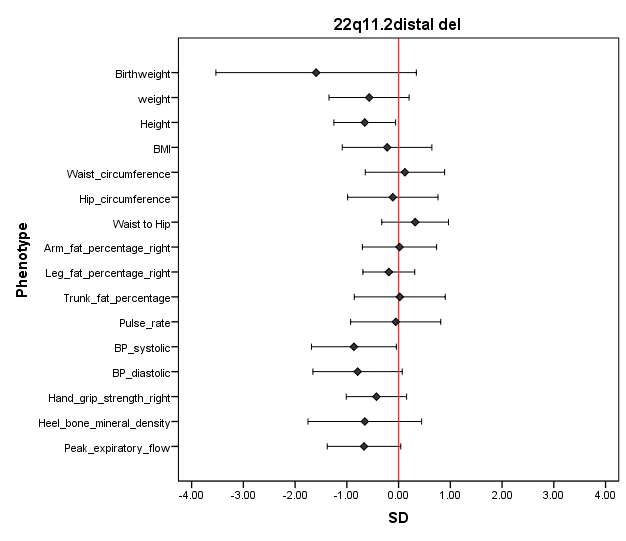

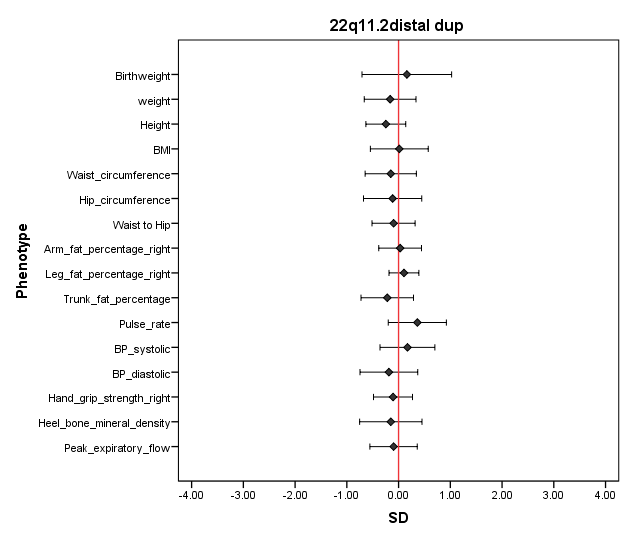

Supplement: Supplementary file 2 — Figure S1. Images of the changes in the physical traits (normalised z-score values) associated with each CNV and their 95% confidence intervals (95%CI). (XLSX 335 kb) (DOCX 1040 kb) [file 12864_2018_5292_MOESM2_ESM.docx]
